# Genetic control of root/shoot biomass partitioning in barley seedlings

**DOI:** 10.3389/fpls.2024.1408043

**Published:** 2024-12-02

**Authors:** Alejandra Cabeza, Ana M. Casas, Beatriz Larruy, María Asunción Costar, Vanesa Martínez, Bruno Contreras-Moreira, Ernesto Igartua

**Affiliations:** Aula Dei Experimental Station, EEAD, CSIC, Zaragoza, Spain

**Keywords:** barley, seedling, biomass partitioning, carbon allocation, plant architecture

## Abstract

The process of allocating resources to different plant organs in the early stage of development can affect their adaptation to drought conditions, by influencing water uptake, transpiration, photosynthesis, and carbon storage. Early barley development can affect the response to drought conditions and mitigate yield losses. A distinct behavior of biomass partitioning between two Spanish barley landraces (SBCC073 and SBCC146) was observed in a previous rhizotron experiment. An RIL population of approximately 200 lines, derived from the cross of those lines, was advanced using speed breeding. We devised an experiment to test if seedling biomass partitioning was under genetic control, growing the seedlings in pots filled with silica sand, in a growth chamber under controlled conditions. After 1 week, the shoot and root were separated, oven dried, and weighted. There were genotypic differences for shoot dry weight, root dry weight, and root-to-shoot ratio. The population was genotyped with a commercial 15k SNP chip, and a genetic map was constructed with 1,353 SNP markers. A QTL analysis revealed no QTL for shoot or root dry weight. However, a clear single QTL for biomass partitioning (RatioRS) was found, in the long arm of chromosome 5H. By exploring the high-confidence genes in the region surrounding the QTL peak, five genes with missense mutations between SBCC146 and SBCC073, and differential expression in roots compared to other organs, were identified. We provide evidence of five promising candidate genes with a role in biomass partitioning that deserve further research.

## 1 Introduction

Photosynthate allocation and partitioning encompass the regulatory processes governing the distribution of fixed carbon within plants. These regulatory mechanisms dictate the allocation of carbon toward storage, intracellular metabolism, or immediate transport to sink tissues. In sink tissues, sugars are allocated toward growth processes of different plant organs (Taiz et al., 2015). On the other hand, partitioning refers to the selective distribution of photosynthates throughout the entire plant. Partitioning mechanisms dictate the amounts of fixed carbon directed toward specific sink tissues. Processes, such as phloem loading and unloading, alongside photosynthate allocation and partitioning, are subjects of significant research interest due to their pivotal roles in enhancing crop productivity (Slafer et al., 2023).

Over the life cycle of the plants, the dynamics of photosynthate allocation result in allometric growth of plant tissues, with an effect on the relative size of the organs and, hence, on the ability to capture the resources from the surrounding environment. Plants devoting more growth to roots invest more in foraging for water and nutrients, whereas plants investing more on shoots and leaves maximize radiation and CO_2_ capture (Siddiqui et al., 2021). Then, to have reproductive and agronomic success, plants must be able to derive an important part of resources toward the reproductive organs, to produce healthy seeds in the largest number possible.

To identify bottlenecks limiting growth and yield, plant physiologists classically take a source-sink perspective. This is a view of growth at the whole-plant scale incorporating mechanistic interactions between physiology, resource allocation, and plant development (White et al., 2016). Sink strength can be defined as the ability of a sink tissue to mobilize photosynthate by itself. It depends on two components: the sink size (total weight of sink tissue) and the sink activity (rate of uptake photosynthates per unit weight of sink tissue). Tissues can be simultaneously source and sink (roots are a source for nitrogen and a sink for carbon). The pull strength of sinks determines the allometric growth. Biomass partitioning between roots and shoots has been identified as an adaptive factor in tree species (Aranda et al., 2010; Voltas et al., 2024), even at seedling stage (Corcuera et al., 2012), with intraspecific variation. In general, populations coming from arid environments show a larger investment in roots than populations coming from humid environments (Lombardi et al., 2021). This feature confers a higher capacity to scavenge for water, thus becoming advantageous for the plants in water-limited environments. Several studies have already looked into biomass partitioning between root and shoot in barley (Bertholdsson and Kolodinska Brantestam, 2009), in some cases finding phenotypic diversity (Voss-Fels et al., 2018; Wang et al., 2021). Some studies looked for QTL in different populations involving a cross of wild and cultivated barley (Arifuzzaman et al., 2014), a wide panel of wild and cultivated barley (Reinert et al., 2016), spring cultivars (Abdel-Ghani et al., 2019), or spring landraces (Khodaeiaminjan et al., 2023). In all these cases, a few QTL for root-to-shoot ratio (calculated in different manners) were found. There are no studies, however, focusing on winter landraces, which make the most abundant barley landrace groups in Spain (Yahiaoui et al., 2008).

Three lines of the Spanish Barley Core Collection (SBCC042, SBCC073, and SBCC146) and three cultivars (Cierzo, Orria, and Scarlett) were tested previously under control and drought conditions for root and shoot growth (Boudiar et al., 2020). This experiment was carried out for 4 weeks using the rhizotrons of the GrowScreen-Rhizo phenotyping platform (Nagel et al., 2012) from the Plant Sciences, Forschungszentrum Jülich GmbH, Germany. It was found that SBCC073 devoted relatively more resources to roots, whereas SBCC146 devoted relatively more resources to shoots. These two lines also showed good field performance, particularly SBCC073, which was the best line of a large set of landraces and modern cultivars tested in a field network in Spain (Yahiaoui et al., 2014).

Our research question is to find out whether genetic variation for biomass partitioning between roots and shoots exists in winter barley landraces from Spain, and to discover QTL that may underlie its genetic control. We will use a biparental barley RIL population that, based on earlier evidence, has the potential to show differences in early allometric growth.

## 2 Materials and methods

### 2.1 Plant material development

The population was developed from an F1 cross between inbred lines SBCC073 and SBCC146. These lines were developed by single seed descent from two Spanish six-rowed landraces, belonging to the two main germplasm groups detected in the Spanish Barley Core Collection (SBCC), using molecular markers (Yahiaoui et al., 2008). SBCC073 comes from the southern part of the country, with warm temperatures and high evapotranspiration, and SBCC146 from cooler areas in the central plateau region. The original cross was performed in 2016. The F1 was multiplied in the field the following year, and the F2 was sown in a growth chamber in the 2018–2019 season. A total of 232 spikes from individual plants were harvested in the F3 plot, to start producing a recombinant inbred line population by single seed descent (SSD). The seed was advanced from F3 to F5 by two generations of speed breeding in a growth chamber following the protocols of Ghosh et al. (2018). The F5 plants were then multiplied in the field in the season 2019–2020. At the end of this process, 195 families were left, which were field multiplied and/or tested in the ensuing seasons. The remaining F6 lines were sown in the field in the 2020–2021 season, for seed multiplication, in plots of two rows, 1-m long, without replication, at the experimental fields of EEAD-CSIC.

### 2.2 Phenotyping test

One hundred and ninety-three genotypes derived from the cross of SBCC073 and SBCC146, and the parents, were tested for root dry weight (RDW), shoot dry weight (SDW), total dry weight (TDW), and root/shoot ratio (RatioRS). Five varieties were used as fillers. We devised a bespoke phenotyping test, for which several sand substrates were evaluated in several tests, until an appropriate type was found. The sand chosen produced barley plants of normal aspect and development and allowed easy washing of the roots. The particularity of this phenotyping test lies in the choice of substrate; since one of the main drawbacks of using commercial substrates is the impossibility of eliminating all traces of soil adhered to the root.

**Figure 1:**
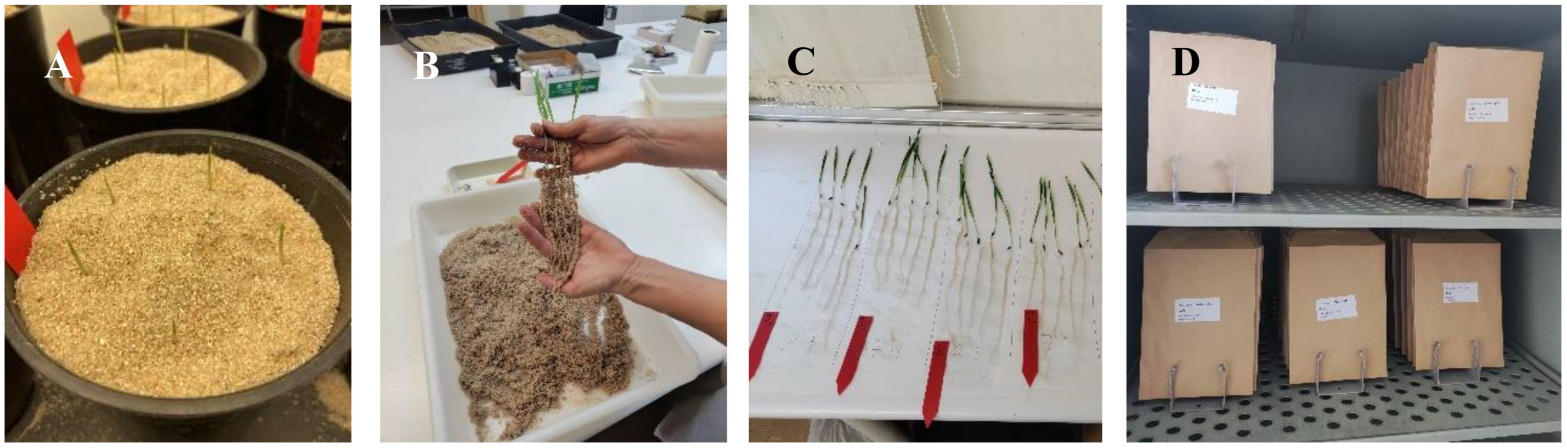
Development of the different experimental phases. **(A)** Pre-germinated seedlings just transplanted to the pot. **(B)** Root extraction. **(C)** Roots and shoot air dried. **(D)** Shoots and roots oven dried.

Seeds were pre-germinated in Petri dishes. After 72 h, five similar size germinated seeds of each line were transferred to pots (18-cm high, 15-cm outer diameter) filled with 2 L of silica sand (0.4–0.8 mm) and irrigated with 100 ml of Hoagland nutrient solution 0.2× (**Figure 1A**). For 1 week, plants developed in a growth chamber under 16-h light/8-h night photoperiod, at 22°C/18°C, 70% humidity, and 250 µmol m^−2^ s^−1^. Pots were irrigated with 50 ml of Hoagland 0.2× twice during the experiment. After 1 week, plants were extracted from pots, and roots were cleaned using low-pressure tap water, carefully (**Figure 1B**). Roots and shoots were air dried during a few hours (**Figure 1C**) and then transferred to paper envelopes. All plants from each pot were kept together in the same envelope (five plants per pot were the experimental unit). Seedlings with abnormal root or shoot development were discarded. Abnormal plants were discarded visually after extracting and cleaning the roots. Extremely short shoot and/or root compared to the other plants of the same genotype were discarded. Roots and shoots were oven dried for 72 h at 70°C (**Figure 1D**) and weighted in a precision scale (Ohaus PR223/E).

The experiment comprised four replications of 200 genotypes each. Four rounds, being 50 genotypes studied in each round, formed each replication. Each round (50 pots, in five trays) was planted and transferred to the growth chamber in a single day. Two or three sowing dates were performed per week. Each genotype was replicated four times (four pots), so a total of 800 pots were used in the experiment. The experimental unit for each genotype was the aggregated value of five seedlings planted in a single pot. Recombinant inbred lines (RIL), checks, and parents (200 genotypes in total) were randomized in five trays, with 10 pots on each tray. Four rounds of five trays conformed one repetition of the whole experiment. Trays were numbered, and their positions inside the growth chamber were consistent across the experiment. When abnormally developed plants were discarded, the data taken from the remaining four plants were multiplied by the appropriate factor to equalize them to the other observations.

### 2.3 Statistical analysis

Statistical analyses were performed, considering growth chamber position, round, repetition, and round nested within repetition as random effects. The variance components for the population and parents were obtained using dummy variables for RILS vs. parents, parents, and RILs as in Piepho et al. (2006). Best linear unbiased estimations (BLUEs) for each RIL were obtained considering genotype as fixed effect. To control for the possible influence of seed size on the traits measured, additional analyses were run with thousand kernel weight (TKW) as covariate. All models were run using ASReml-R (Butler et al., 2017). Means and confidence intervals were obtained using the R package “predictmeans” (Luo et al., 2018).

### 2.4 QTL analysis

DNA was extracted from 7-day-old individual seedlings using the EchoLUTION Plant DNA kit (BioEcho, GmbH). Genotyping was performed with a proprietary 15k Barley Infinium SNP array by SGS Institute Fresenius GmbH, TraitGenetics Section. A genetic map was then constructed for 193 F5 RIL lines with JoinMap 4, assigning 3,566 SNP markers to seven linkage groups. After filtering co-segregating markers, 1,353 SNPs with unique map position were used for QTL analysis (**Supplementary Table S1**), with the software Genstat Release 22.1 (VSN International, 2022). For each trait, QTL analysis was carried out using composite interval mapping, calculating genetic predictors every 3 cM, setting the minimum distance for QTL peaks to 20 cM, and the minimum distance between cofactors to 30 cM. The procedure was run iteratively until the number of QTLs detected stabilized. The Li and Ji (2005) method was used to estimate a 5% genome-wide significance threshold for the −log_10_ p-values. For each marker, physical position in the Morex V3 reference genome (Mascher et al., 2021) was retrieved from Barleymap (Cantalapiedra et al., 2015).

### 2.5 Searching for candidate genes

Gene models in the reference genome Morex V3 (Mascher et al., 2021), within the QTL confidence interval, were retrieved using Barleymap (Cantalapiedra et al., 2015). Then, genes were classified into high- (HC) or low-confidence classes, and the HC genes with design of exome capture targets were identified in BARLEX (Colmsee et al., 2015). The subset of exome-captured HC genes was further reduced to those genes showing SNP polymorphisms between exome capture data of the parents, which was available in-house (Casas et al., 2018). Exome sequencing was performed previously according to the methods described by Mascher et al. (2013). DNA sequencing, made at CNAG (Centro Nacional de Análisis Genómico, Barcelona), and data analysis were performed as described by Cantalapiedra et al. (2016). Expression data from a selected set of genes was retrieved from the Barley Expression Database (Li et al., 2023). The selected genes were clustered according to their expression in informative experiments and tissues from the expression database using R function heatmap.2 in package *gplots* (Warnes et al., 2016). Presence of the identified polymorphisms was validated by comparison to the SBCC073 transcriptome (Cantalapiedra et al., 2017) and the barley pangenome (Jayakodi et al., 2020). SIFT scores of non-synonymous protein mutations were retrieved from Ensembl Plants using recipe 8 from Contreras-Moreira et al. (2022). Collinear pangene clusters containing gene models of interest from MorexV3 and the other accessions in the pangenome, built with version 11012024 of GET_PANGENES (Contreras-Moreira et al., 2023), were retrieved from https://eead-csic-compbio.github.io/barley_pangenes. Alignment of amino acid sequences of candidate genes were plotted with NCBI MSA Viewer 1.25.0. We used the Frequency-Based Differences method, which assigns scores to bases based on their representation in the column’s frequency profile.

## 3 Results

### 3.1 Genotypic differences in seedling growth

**Figure 2:**
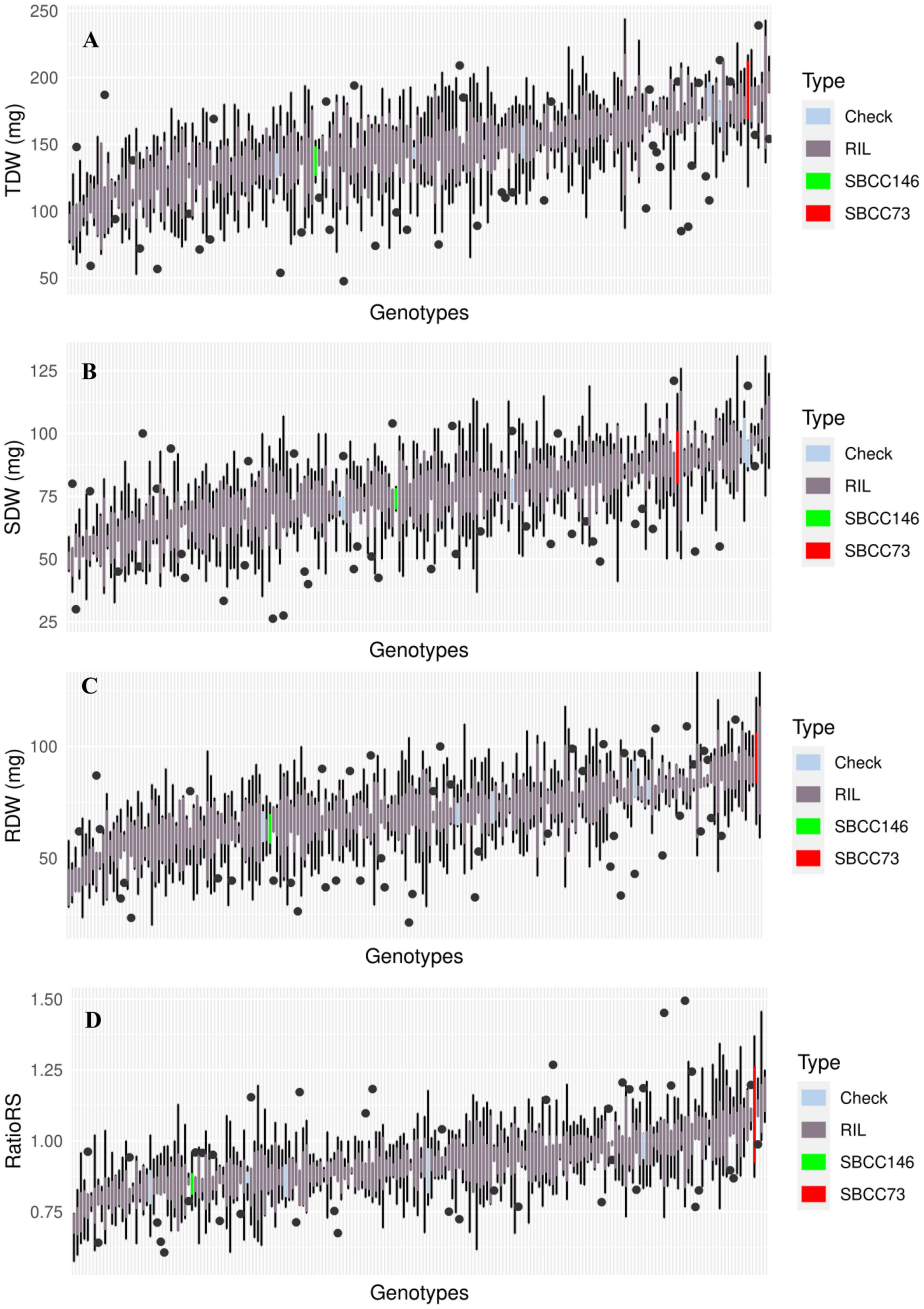
Boxplot representing median, minimum, and maximum of **(A)** TDW, **(B)** SDW, **(C)** RDW, and **(D)** RatioRS of the whole set of genotypes tested in the experiment. In red, parent SBCC073; in green, parent SBCC0146; in dark gray, a set of RIL; and in light blue, five genotypes as checks.

The two parents showed quite divergent values for all traits, and the distributions of the variables for the population showed a smooth quantitative variation, when lines were ordered from lowest to highest values, for all four traits considered (**Figure 2**, **Supplementary Table S2**).

The analyses of variance revealed significant genotypic differences for all four traits considered, for the population lines, and for parents (**Table 1**). Parent SBCC073 showed higher RDW and SDW than SBCC146, although the difference was not significant. SBCC073 turned out to present the highest RDW mean value of the whole experiment. The parents of the population only showed differences in RatioRS, even when TKW was used as a covariate (**Supplementary Figure S1**), confirming the results of Boudiar et al. (2020). Considering these traits, SBCC073 exhibits more RDW, SDW, TDW, and RatioRS; therefore, it appears to be a genotype that invests more photosynthetic assimilates in both parts when compared to the other parent of the population, SBCC146.

**Table 1:** Analyses of variance results.

Wald statistic	Source of variation		
	Parents	RILS vs. parents	RILS
DF	1	1	192
TDW	1.43	1.73	323.82***
SDW	0.26	0.93	318.92***
RDW	3.14	2.42	328.87***
RatioRS	6.21*	1.64	346.13***
TDW_SS	0.30	0.01	267.48***
SDW_SS	0.11	0.037	267.23***
RDW_SS	0.66	0.048	264.33***
RatioRS_SS	4.38*	0.50	336.02***

The genotype source of variation was broken down into the corresponding terms for population RILs, parents, and the contrast between RILs and parents.

_SS, analyses taking into account seed size (thousand kernel weight) as covariate.

p-value < 0.05 *, 0.01 **, 0.001 ***.

### 3.2 QTL analysis

**Figure 3:**
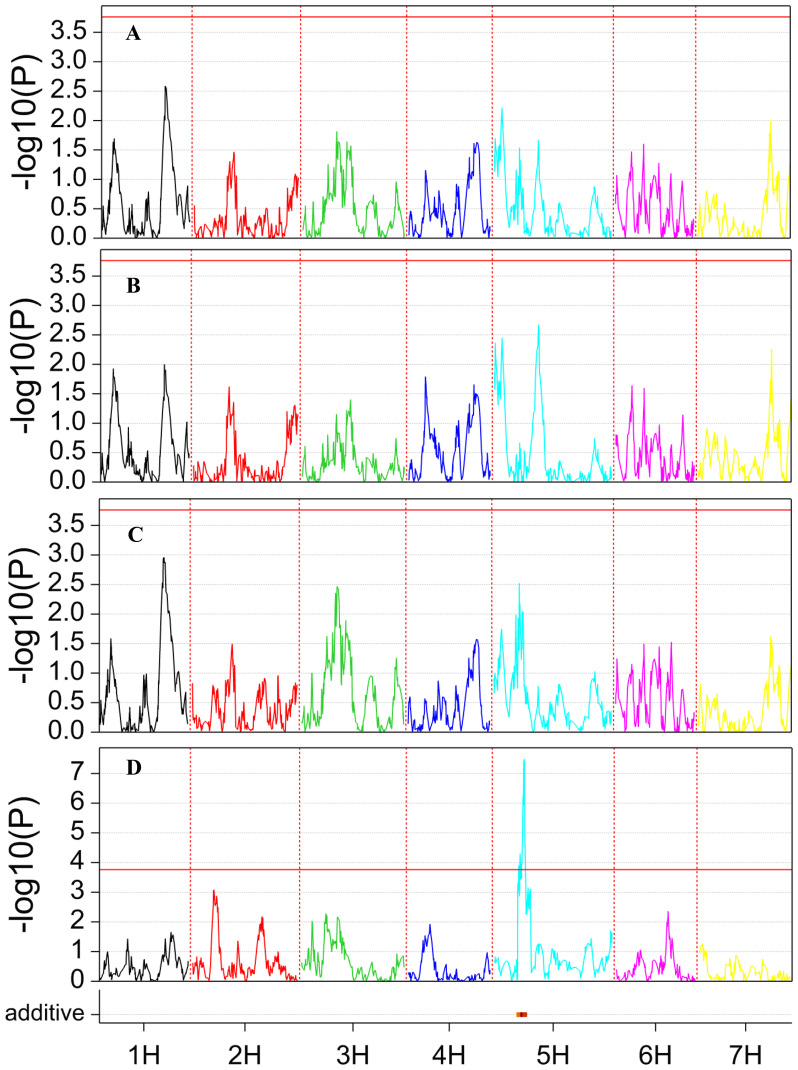
QTL scan for the traits recorded in this study. **(A)** TDW, **(B)** SDW, **(C)** RDW, and **(D)** RatioRS. At the bottom of the graphs, a red dash indicates the presence of a QTL for which SBCC073 contributes the allele with the larger value.

A single QTL was detected for the RatioRS trait, located on the long arm of chromosome 5H at 66.6 cM (400,664,453 bp) (**Figure 3**), explaining 15.2% of the phenotypic variance. The allele of SBCC073 led to increasing the ratio by 0.062. This QTL was independent of seed size, as it was also detected when TKW was used as covariate. Interestingly, no other QTL was detected for any other traits, although there were genotypic differences for all of them (**Table 1**) suggesting that the partitioning QTL detected was instrumental in the phenotypic differences found for seedling architecture.

### 3.3 Candidate genes

The peak marker for the RatioRS QTL was BOPA2_12_10725 located within gene HORVU.MOREX.r3.5HG0475170. The QTL confidence interval ranged from 62.6 to 66.9 cM, or 350,508,439 to 403,211,990 bp in the reference genome. The region contains 589 gene models, with 270 HC genes, including 189 with exome capture data. For each of those genes, inspection of putative variants between SBCC146 and SBCC073 recognized 102 genes without variants, 43 genes with polymorphisms outside the coding region, 22 genes with synonymous SNPs, and 22 genes with non-synonymous SNPs. We examined the annotated gene descriptions and constructed a heat map, based on expression data, for 24 genes, including those with non-synonymous SNPs (**Supplementary Tables S3 and S4**; **Supplementary Figure S2**). SBCC073 displayed missense mutations in four genes that showed higher expression in roots than in other tissues making them good candidate genes (**Supplementary Table S5**). Alignment of amino acid sequences for the candidate genes in the barley pangenome revealed natural variation for all of them (**Supplementary Figure S3**). Thus, five polymorphisms were found in gene HORVU.MOREX.r3.5HG0473970, Glycosyltransferase (D344V, R362Q, R413H, K430E, and V469A). The variants identified in SBCC073 were also found in pangenome genotypes, such as HOR13942 or RGT Planet, so they are likely true variants. Also, seven polymorphisms were found in model HORVU.MOREX.r3.5HG0474460, Protein kinase, putative (G14A, W147R, H149S, S159L, V184A, F281L, and G757S). The same variants were identified in the pangenome genotype HOR13942. Nine polymorphisms were identified in gene HORVU.MOREX.r3.5HG0474880, Leguminosin group485 secreted peptide (Y25H, G26C, I46L, V63A, V64M, A68T, V90L, R138P, and E153A), all of them in common with pangenome genotype HOR13942. It is worth mentioning another gene (HORVU.MOREX.r3.5HG0471560, Receptor kinase), in which SBCC073 showed a missense polymorphism (F7L), although in this case, our results do not cover the complete sequence of the gene. The other parent of the population, SBCC146, had missense polymorphisms in another gene, HORVU.MOREX.r3.5HG0474810, Receptor-like protein kinase, which showed lower expression in roots than in other tissues. SBCC146 and SBCC073 differed in three polymorphisms (P204T, P277L, and A422V), and both genotypes carried the alternative allele in two other cases (S324P and N599T). Those five variants, identified in SBCC146, were all present in pangenome genotype Igri. Therefore, this gene is also a good candidate yielding in total five candidates.

## 4 Discussion

The results found in this experiment indicate a difference in assimilate partitioning in young seedlings of the RIL population between two Spanish landraces belonging to different genetic groups, which prevailed in areas with contrasting agro-climatic conditions. The parents follow a similar root growth trend as reported by Boudiar et al. (2020), with more vigorous root growth for SBCC073, but not for shoot growth, which was similar for the two genotypes. The differences in experimental set up and duration of the experiment are likely causing this discrepancy. Both experiments support that SBCC073 allocates more biomass to root tissue.

Is it more beneficial for a plant to invest more carbon resources to root tissue or to an aerial part at the first stage of development? During the first stages of development, crops need to be well established acquiring enough water and nutrients, and the root system is one of the main organs that requires carbon investment. On the other hand, plants need to produce energy through photosynthesis, so increasing shoots will derive into more photosynthetic tissue and a more vigorous canopy development. The difference found between these landraces actually bodes well with their geographic origin. SBCC073 is representative of a genetic group coming from dryer and warmer areas than those where the group of SBCC146 was established (Contreras-Moreira et al., 2019). We hypothesize that the presence of an allele favoring biomass allocation to roots in a barley from dry areas can be an adaptive trait. This hypothesis should be tested in more landrace materials of known origins. Studies comparing root system architecture at seedling and adult stages suggested that the root system architecture does not correlate well at both developmental phases (Maccaferri et al., 2016; Williams et al., 2022). This means that the root architecture traits observed at seedling stage are not expected to carry over at adult stage and that it is necessary to confirm the biomass partitioning trait detected in this population at adult stage.

Moreover, it will be interesting to test how this population modulates biomass allocation under drought stress, at seedling and adult stages, since plants are able to modify its phenotype in response to biotic and abiotic environmental signals (Poorter et al., 2012). A previous study (Boudiar et al., 2020) suggested that SBCC073 decreased RDW by 43% and SBCC146 by only 16%, under drought, in a 4-week experiment in rhizotrons.

A QTL for the root/shoot ratio was identified on chromosome 5H, with SBCC073 contributing the favorable allele. The QTL peak marker was at 66.6 cM (400.66 Mb), with a confidence interval ranging from 350.50 to 403.21 Mbp in the reference genome. This region is proximal to the one (between 68.78 and 320.04 Mbp on chromosome 5H) identified by Wonneberger et al. (2023) in modern European spring two-rowed barleys. The authors suggested that this haplotype probably originated from Northern Africa. Indeed, the six-rowed Spanish landrace SBCC073 mostly shared the RGT Planet haplotype in that region (132 identical SNPs out of 137, from 67.6 to 319.9 Mbp), but was different at the QTL region. Different genome-wide association studies have searched for QTL for root-to-shoot ratio in barley using plastic pots (Reinert et al., 2016), filter rolls (Abdel-Ghani et al., 2019), or germination pouches (Khodaeiaminjan et al., 2023). Those studies looked at biomass partitioning in plants older than ours, and identified QTL for this trait on chromosome 5H but distal to our QTL region (475–495 Mbp), pointing to a different region in the genome.

We searched for putative candidate genes in the region, focusing on those with differential expression between roots and shoots in other transcriptomic studies, and containing SNP polymorphisms in the coding region. Two of them, a UDP-glycosyltransferase (HORVU.MOREX.r3.5HG0473970), and a gene annotated as Leguminosin group485 secreted peptide (HORVU.MOREX.r3.5HG0474880) showed higher expression in root samples, and both of them accumulated several missense variants between the parents of the population. SBCC073 shared those variants with the pangenome genotype “HOR13942,” a landrace originating from Baeza, Southern Spain, close to the collection site of SBCC073. UDP-glycosyltransferases glycosylate small molecules such as phytohormones, secondary metabolites, and xenobiotics (Gharabli et al., 2023). A rice UDP-glycosyltransferase was identified as QTL regulating grain size by modulating cell proliferation and expansion indirectly affecting auxin levels (Dong et al., 2020). Cytokinin glucosyltransferases have also been identified as key regulators of cytokinin homeostasis, with potential value for wheat breeding (Chen et al., 2021). A gene annotated as Leguminosin group458 secreted protein was the most upregulated gene in barley leaves during the recovery phase after a drought treatment (Paul et al., 2023). Nevertheless, that gene is located in a different position on chromosome 5H (539.41 Mbp). We did not identify orthologs in *Arabidopsis* or rice, but a BLASTp of the transcript corresponding to the gene identified in this study had its best match [100% identity, expected value (E-value) of 3e−93] to a protein PELPK1-like from barley (XP_044947259.1). An *Arabidopsis thaliana* PELPK1 is a cell wall protein required for normal germination and growth (Rashid and Deyholos, 2011). Other gene annotated as Protein kinase, putative (HORVU.MOREX.r3.5HG0474460), an LRR receptor-like protein kinase, was highly expressed in root samples of different transcriptomic studies. Li et al. (2020), investigating the dynamics of C/N-nutrient-related phosphorylation signals in *A. thaliana*, identified related proteins. A Brassinosteroid-like receptor kinase (BRL1-like, HORVU.MOREX.r3.5HG0471560) was also included in the confidence interval of the QTL, although we only identified a tolerated missense variant in that gene. Therefore, we have less evidence in this case to be declared as a candidate gene, although it is still worth mentioning due to the relevance of the gene family. It has been shown that brassinosteroids affect barley root growth (Kartal et al., 2009), and a missense mutation in a BR receptor protein is associated with the semi-dwarf “uzu” mutation in barley (Chono et al., 2003). In rice, Nakamura et al. (2006) observed a high expression of the *OsBRL1* and *OsBRL3* genes in roots and suggested that they may be involved in BR perception in the roots. SBCC146 showed two missense variants in another gene, annotated as Receptor-like protein kinase (HORVU.MOREX.r3.5HG0474810), highly expressed in shoots. Receptor-like kinases are signaling proteins implicated in the regulation of development and stress responses (Morillo and Tax, 2006; Vij et al., 2008; Gish and Clark, 2011). Nevertheless, we cannot rule out that other genes in the interval are responsible for the trait. The results should be confirmed performing the experiment for a longer period validating the polymorphisms identified and testing gene expression of the candidate genes in this population.

We have identified genetic diversity for biomass partitioning between shoots and roots at the seedling stage in barley landraces coming from distinct agro-ecological regions, with potential adaptive meaning. The genetic control of partitioning seems to be a single gene, and we provide genomic evidence for five possible candidate genes that deserve further research, evaluating the performance of contrasting genotypes at seedling stage extending to the growth period, and at adult stage in the field.

## Data Availability

The exome capture dataset utilized in this study is available at the European Nucleotide Archive, accession PRJEB73755.
