# Supplementary material for: Genetic control of root/shoot biomass partitioning in barley seedlings
Source: Front Plant Sci. 2024 Dec 2;15:1408043. doi: 10.3389/fpls.2024.1408043 (PMC11646724; doi:10.3389/fpls.2024.1408043)
Supplement: Supplementary file 1 [file DataSheet1.pdf]

Supplementary Material

1 Supplementary Figures

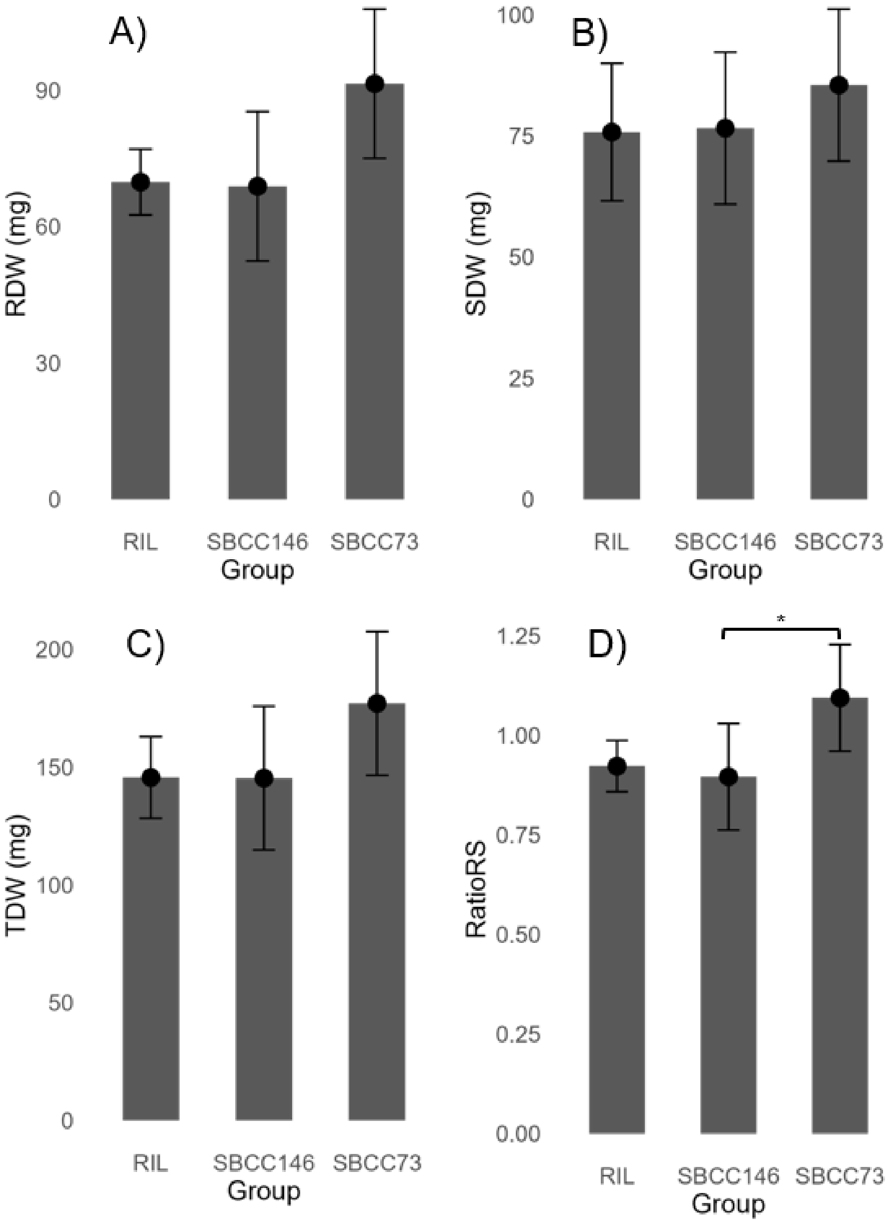

Supplementary Figure 1. Means of **(A)** RDW, **(B)** SDW, **(C)** TDW and **(D)** RatioRS. Bars represent the mean and CI at 95%. RatioRS is statistically significant between the parents of the population.

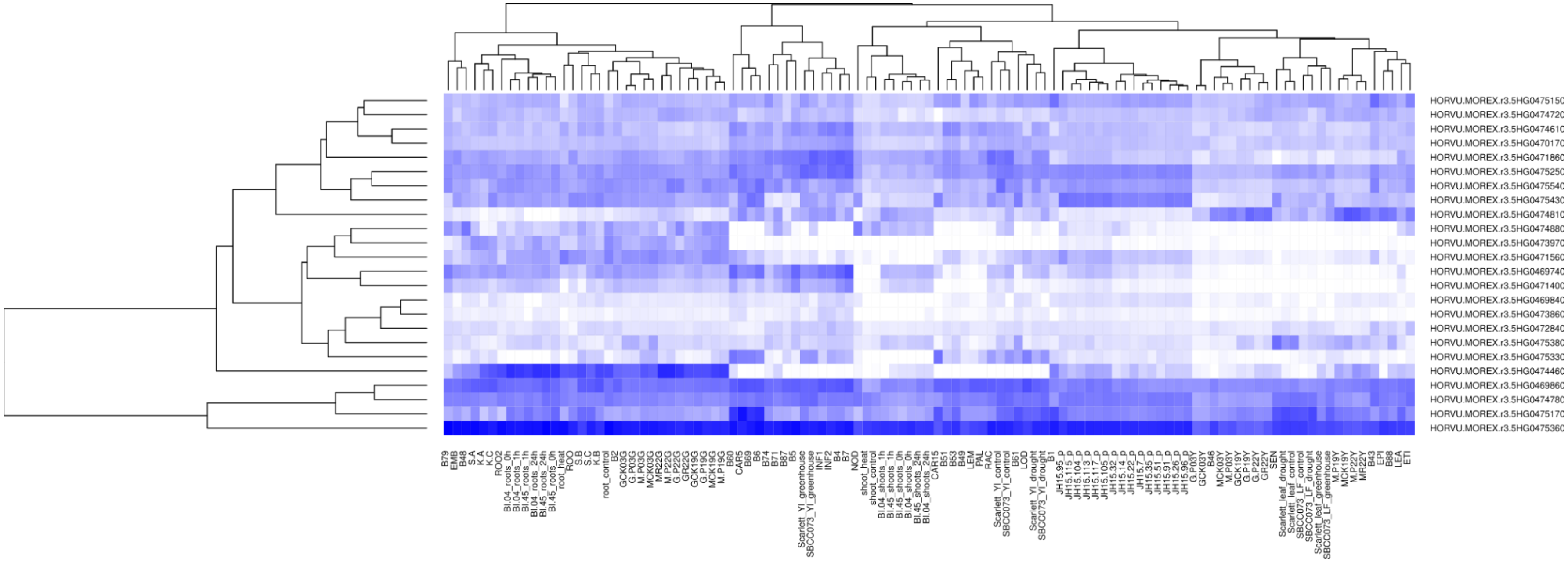

Supplementary Figure 2. Clustering of differentially expressed genes in a range of tissues and treatments. Data collected from 8 transcriptome projects reported in Table S5.2.

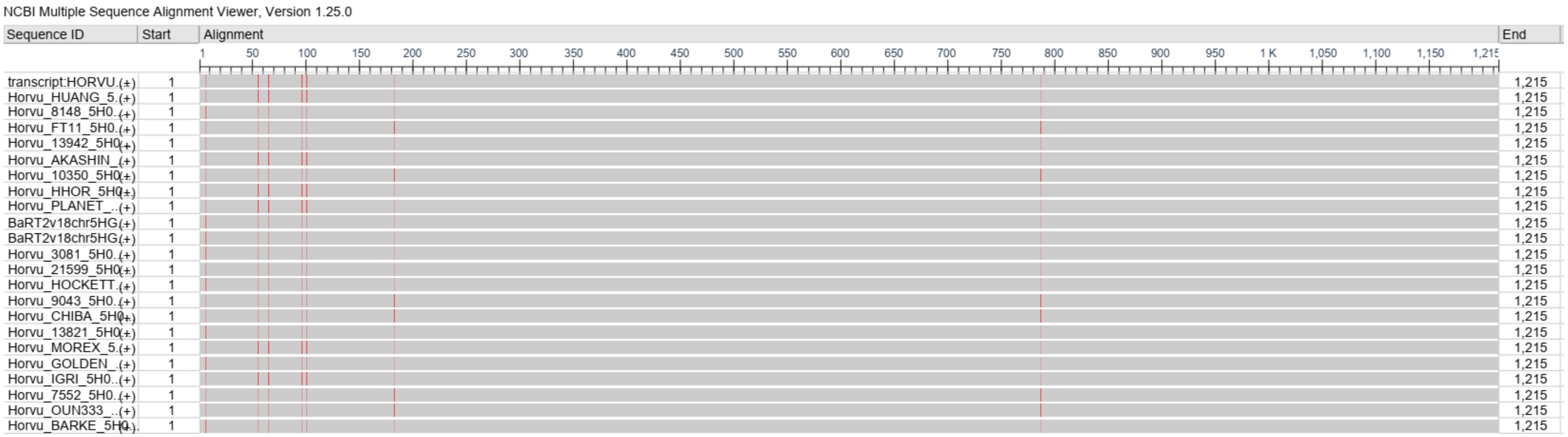

Supplementary Figure 3. Alignment of amino acid sequences for gene HORVU.MOREX.r3.5HG0471560. Bases that occur infrequently are highlighted darker than others. Positions that contain any degree of mismatch are also highlighted in the anchor or consensus row.

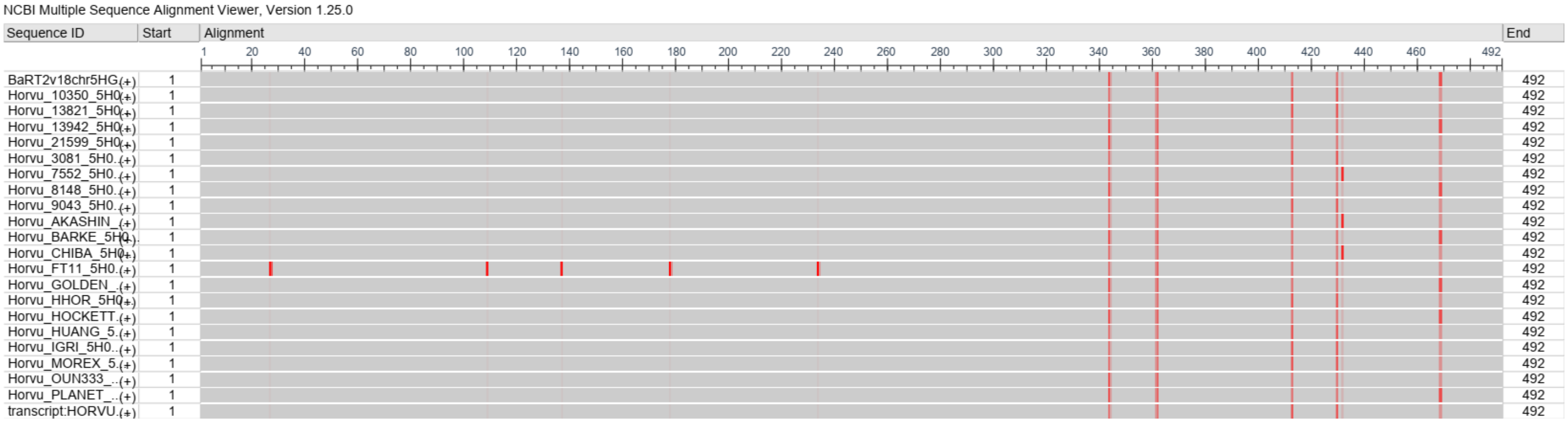

Supplementary Figure 3 (continue). Alignment of amino acid sequences for gene HORVU.MOREX.r3.5HG0473970.

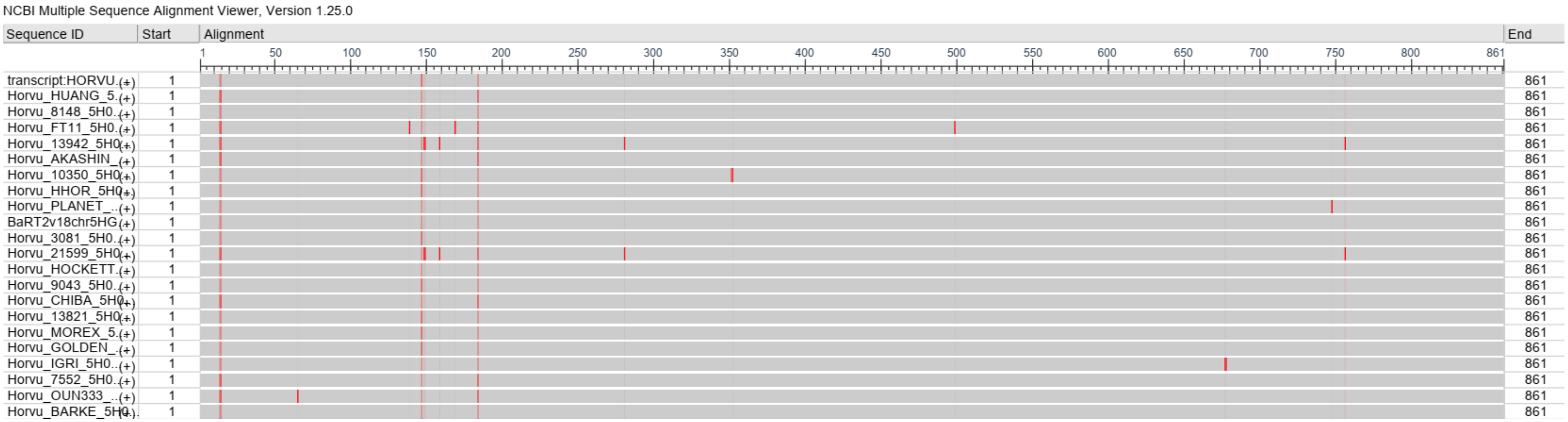

Supplementary Figure 3 (continue). Alignment of amino acid sequences for gene gene-HORVU.MOREX.r3.5HG0474460.

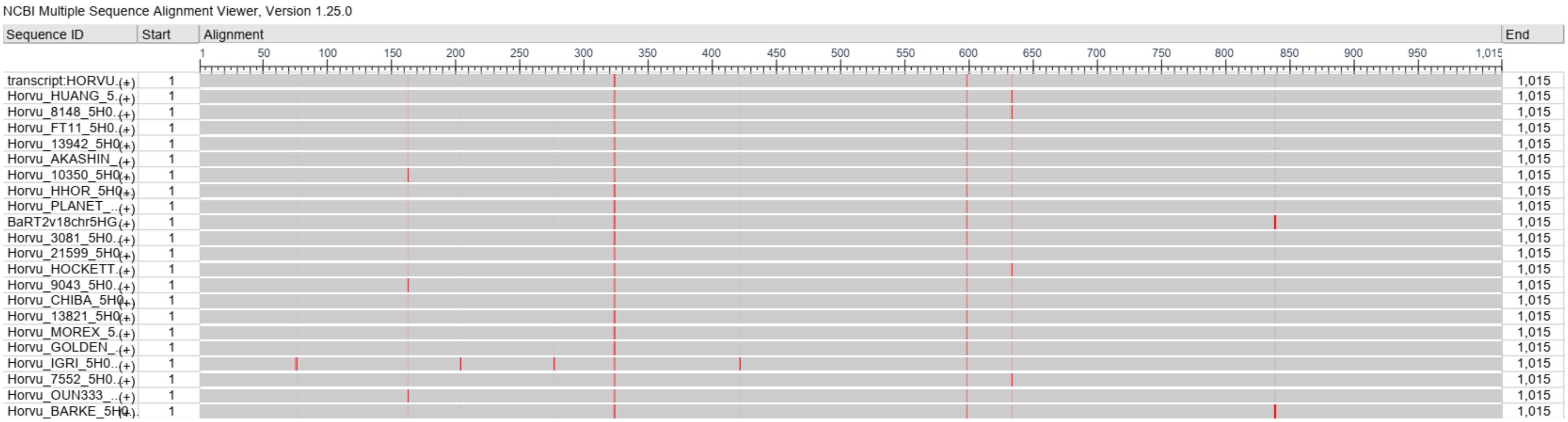

Supplementary Figure 3 (continue). Alignment of amino acid sequences for gene gene-HORVU.MOREX.r3.5HG0474810.

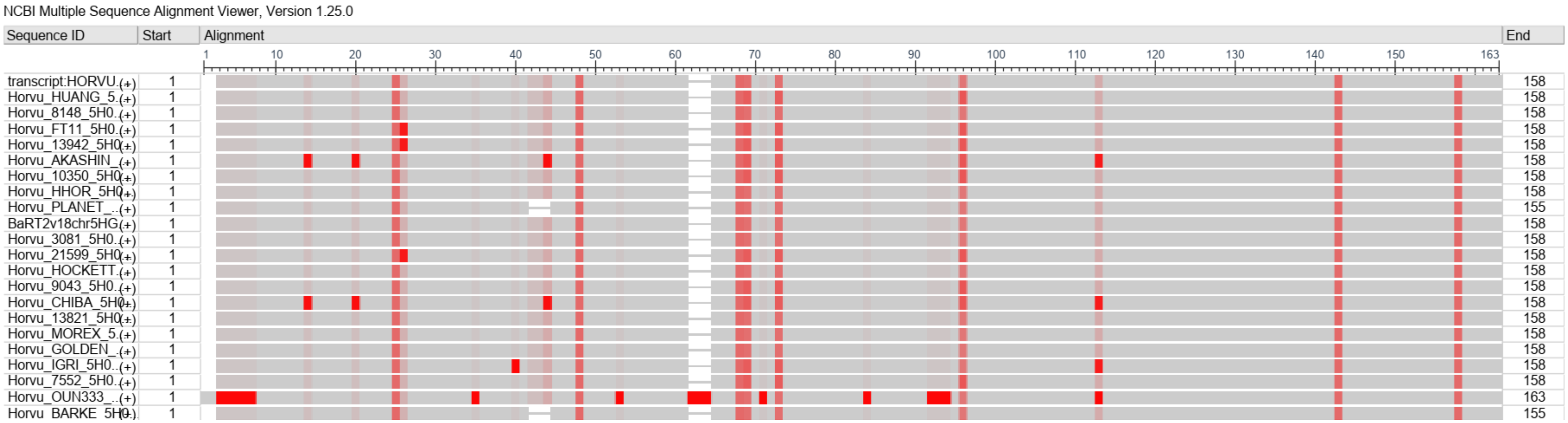

Supplementary Figure 3 (continue). Alignment of amino acid sequences for gene gene-HORVU.MOREX.r3.5HG0474880.
